# 1-[Bis(4-fluoro­phen­yl)meth­yl]-4-[2-(naphthalen-2-yl­oxy)eth­yl]piperazine

**DOI:** 10.1107/S1600536812024129

**Published:** 2012-05-31

**Authors:** Yan Zhong, Bin Wu

**Affiliations:** a School of Chemistry and Chemical Engineering, Southeast University, Sipailou No. 2 Nanjing, Nanjing 210096, People’s Republic of China; b School of Pharmacy, Nanjing Medical University, Hanzhong Road No. 140 Nanjing, Nanjing 210029, People’s Republic of China

## Abstract

In the title mol­ecule, C_29_H_28_F_2_N_2_O, the piperazine ring adopts a chair conformation with the pendant N—C bonds in equatorial orientations. The conformation of the N—C—C—O linkage is *gauche* [torsion angle = −64.6 (4)°] and the dihedral angle between the fluoro­benzene rings is 64.02 (15)°.

## Related literature

For related structures and background to 1-[bis­(4-fluoro­phen­yl)meth­yl]piperazine derivatives, see: Wu *et al.* (2008 ▶); Dayananda *et al.* (2012 ▶); Dai *et al.* (2012 ▶).
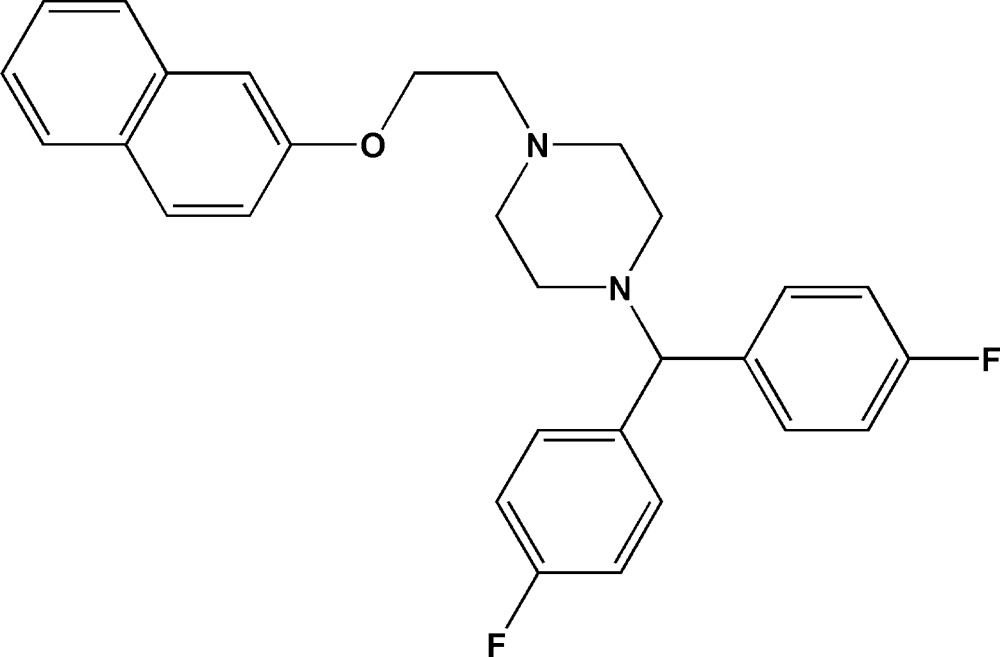


## Experimental

### 

#### Crystal data

C_29_H_28_F_2_N_2_O
*M* *_r_* = 458.53
Monoclinic, 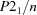

*a* = 10.416 (2) Å
*b* = 16.870 (3) Å
*c* = 14.311 (3) Å
β = 100.57 (3)°
*V* = 2472.0 (9) Å^3^
*Z* = 4
Mo *K*α radiation
μ = 0.09 mm^−1^
*T* = 293 K
0.30 × 0.20 × 0.10 mm

#### Data collection

Enraf–Nonius CAD-4 diffractometer
4802 measured reflections
4541 independent reflections
2743 reflections with *I* > 2σ(*I*)
*R*_int_ = 0.087
3 standard reflections every 200 reflections intensity decay: 1%

#### Refinement

*R*[*F* ^2^ > 2σ(*F* ^2^)] = 0.058
*wR*(*F* ^2^) = 0.182
*S* = 1.01
4541 reflections
307 parameters
4 restraints
H-atom parameters constrained
Δρ_max_ = 0.26 e Å^−3^
Δρ_min_ = −0.22 e Å^−3^

### 

Data collection: *CAD-4 EXPRESS* (Enraf–Nonius, 1989 ▶); cell refinement: *CAD-4 EXPRESS*; data reduction: *XCAD4* (Harms & Wocadlo, 1995 ▶); program(s) used to solve structure: *SHELXS97* (Sheldrick, 2008 ▶); program(s) used to refine structure: *SHELXL97* (Sheldrick, 2008 ▶); molecular graphics: *SHELXL97*; software used to prepare material for publication: *SHELXL97*.

## Supplementary Material

Crystal structure: contains datablock(s) I, global. DOI: 10.1107/S1600536812024129/hb6811sup1.cif

Structure factors: contains datablock(s) I. DOI: 10.1107/S1600536812024129/hb6811Isup2.hkl

Supplementary material file. DOI: 10.1107/S1600536812024129/hb6811Isup3.cml

Additional supplementary materials:  crystallographic information; 3D view; checkCIF report

## Figures and Tables

**Fig. 1.**
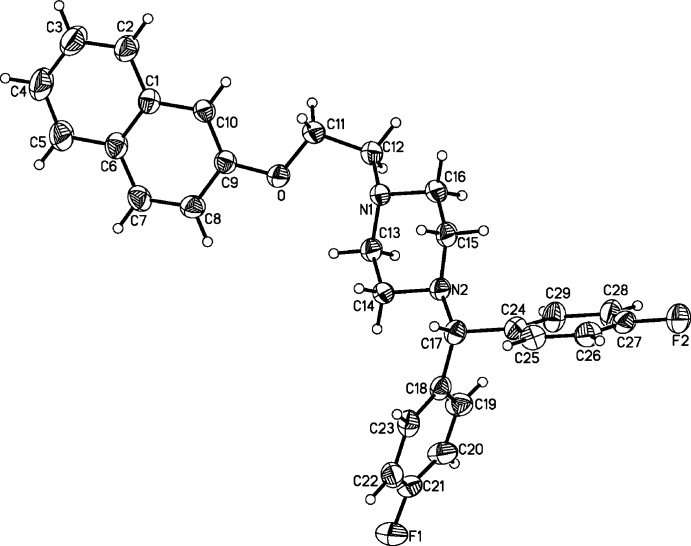
The molecular structure of (I) with displacement ellipsoids for non-H drawn at 70% probability level.

## supplementary crystallographic information

### Comment

As a continuation of our study of 1-(bis(4-fluorophenyl)methyl)piperazine derivatives (Wu *et al.*, 2008; Dai *et al.*, 2012), we present here the title compound (I). In (I) (Fig. 1), all bond lengths and angles are normal and correspond to those observed in related compounds (Dai *et al.*, 2012). The piperazine ring adopts a chair conformation with puchering parameters Q = 0.590 (3), Theta = 176.9 (3), Phi = 10 (5). The dihedral angle between the fluorobenzene rings is 64.02 (15).

### Experimental

A mixture of 2-(2-bromoethoxy)naphthalene (10 mmol), 1-(bis(4-fluorophenyl)methyl)piperazine (15 mmol) and triethylamine (5 ml) were mixed along with 40 ml acetonitrile and then refluxed for about 24 h. The progress of the reaction was monitored by TLC. After confirming that the reaction was completed, the solvent was removed under reduced pressure. The resultant mixture was cooled. The solid, 1-(bis(4-fluorophenyl)methyl)-4-(2-(naphthalen-2-yloxy)ethyl)piperazine obtained was filtered and was recrystallized from ethanol. Colorless blocks were grown from ethyl acetate:hexane (2:1) solution by a slow evaporation at room temperature.

### Refinement

The C-bound H-atoms were included in calculated positions and treated as riding atoms: C—H = 0.93, 0.97 and 0.98 Å for CH(aromatic), CH_2_ and CH(methine) H-atoms, respectively, with *U*ĩso~(H) = 1.2 *U*~eq~ of the carrier atom.

### Crystal data

**Table:**

C_29_H_28_F_2_N_2_O	*F*(000) = 968
*M**_r_* = 458.53	*D*_x_ = 1.232 Mg m^−^^3^
Monoclinic, *P*2_1_/*n*	Mo *K*α radiation, λ = 0.71073 Å
*a* = 10.416 (2) Å	Cell parameters from 25 reflections
*b* = 16.870 (3) Å	θ = 10–13°
*c* = 14.311 (3) Å	µ = 0.09 mm^−^^1^
β = 100.57 (3)°	*T* = 293 K
*V* = 2472.0 (9) Å^3^	Block, colorless
*Z* = 4	0.30 × 0.20 × 0.10 mm

### Data collection

**Table:**

Enraf–Nonius CAD-4 diffractometer	*R*_int_ = 0.087
Radiation source: fine-focus sealed tube	θ_max_ = 25.4°, θ_min_ = 1.9°
Graphite monochromator	*h* = 0→12
ω/2θ scans	*k* = 0→20
4802 measured reflections	*l* = −17→16
4541 independent reflections	3 standard reflections every 200 reflections
2743 reflections with *I* > 2σ(*I*)	intensity decay: 1%

### Refinement

**Table:**

Refinement on *F*^2^	Primary atom site location: structure-invariant direct methods
Least-squares matrix: full	Secondary atom site location: difference Fourier map
*R*[*F*^2^ > 2σ(*F*^2^)] = 0.058	Hydrogen site location: inferred from neighbouring sites
*wR*(*F*^2^) = 0.182	H-atom parameters constrained
*S* = 1.01	*w* = 1/[σ^2^(*F*_o_^2^) + (0.1*P*)^2^ + 0.2*P*] where *P* = (*F*_o_^2^ + 2*F*_c_^2^)/3
4541 reflections	(Δ/σ)_max_ < 0.001
307 parameters	Δρ_max_ = 0.26 e Å^−^^3^
4 restraints	Δρ_min_ = −0.22 e Å^−^^3^

### Special details

**Table:**

Geometry. All e.s.d.'s (except the e.s.d. in the dihedral angle between two l.s. planes) are estimated using the full covariance matrix. The cell e.s.d.'s are taken into account individually in the estimation of e.s.d.'s in distances, angles and torsion angles; correlations between e.s.d.'s in cell parameters are only used when they are defined by crystal symmetry. An approximate (isotropic) treatment of cell e.s.d.'s is used for estimating e.s.d.'s involving l.s. planes.
Refinement. Refinement of *F*^2^ against ALL reflections. The weighted *R*-factor *wR* and goodness of fit *S* are based on *F*^2^, conventional *R*-factors *R* are based on *F*, with *F* set to zero for negative *F*^2^. The threshold expression of *F*^2^ > σ(*F*^2^) is used only for calculating *R*-factors(gt) *etc*. and is not relevant to the choice of reflections for refinement. *R*-factors based on *F*^2^ are statistically about twice as large as those based on *F*, and *R*- factors based on ALL data will be even larger.

### Fractional atomic coordinates and isotropic or equivalent isotropic displacement parameters (Å^2^)

**Table:**

	*x*	*y*	*z*	*U*_iso_*/*U*_eq_	
O	0.27757 (19)	0.41478 (11)	0.47099 (15)	0.0661 (6)	
N1	0.2649 (2)	0.58274 (13)	0.52960 (17)	0.0575 (6)	
F1	0.90854 (19)	0.59295 (13)	0.96914 (15)	0.0967 (7)	
C1	0.1204 (3)	0.22487 (16)	0.3853 (2)	0.0542 (7)	
F2	0.4277 (2)	1.05347 (11)	0.74130 (15)	0.0922 (7)	
N2	0.4063 (2)	0.68238 (13)	0.67855 (16)	0.0528 (6)	
C2	0.0148 (3)	0.19302 (19)	0.3205 (2)	0.0721 (9)	
H2A	−0.0448	0.2268	0.2840	0.087*	
C3	−0.0006 (4)	0.1129 (2)	0.3110 (3)	0.0988 (13)	
H3A	−0.0707	0.0927	0.2679	0.119*	
C4	0.0860 (4)	0.0619 (2)	0.3644 (4)	0.1085 (15)	
H4A	0.0740	0.0075	0.3572	0.130*	
C5	0.1889 (4)	0.0902 (2)	0.4273 (3)	0.0888 (11)	
H5A	0.2464	0.0549	0.4633	0.107*	
C6	0.2100 (3)	0.17243 (17)	0.4390 (2)	0.0613 (8)	
C7	0.3172 (3)	0.20455 (18)	0.5024 (2)	0.0654 (8)	
H7A	0.3758	0.1705	0.5394	0.078*	
C8	0.3365 (3)	0.28384 (18)	0.5104 (2)	0.0625 (8)	
H8A	0.4091	0.3037	0.5512	0.075*	
C9	0.2463 (3)	0.33609 (16)	0.45675 (19)	0.0534 (7)	
C10	0.1397 (3)	0.30770 (16)	0.3968 (2)	0.0523 (7)	
H10A	0.0792	0.3428	0.3634	0.063*	
C11	0.1892 (3)	0.47098 (17)	0.4185 (2)	0.0688 (9)	
H11A	0.1065	0.4697	0.4405	0.083*	
H11B	0.1731	0.4573	0.3516	0.083*	
C12	0.2474 (3)	0.55314 (17)	0.4322 (2)	0.0694 (9)	
H12A	0.3315	0.5528	0.4123	0.083*	
H12B	0.1912	0.5896	0.3909	0.083*	
C13	0.3933 (3)	0.56131 (17)	0.5841 (2)	0.0598 (8)	
H13A	0.4607	0.5803	0.5511	0.072*	
H13B	0.4003	0.5040	0.5885	0.072*	
C14	0.4152 (3)	0.59602 (16)	0.6829 (2)	0.0569 (7)	
H14A	0.3503	0.5754	0.7171	0.068*	
H14B	0.5008	0.5806	0.7170	0.068*	
C15	0.2747 (3)	0.70173 (17)	0.6280 (2)	0.0624 (8)	
H15A	0.2635	0.7588	0.6257	0.075*	
H15B	0.2106	0.6795	0.6619	0.075*	
C16	0.2528 (3)	0.66947 (17)	0.5293 (2)	0.0648 (8)	
H16A	0.1663	0.6844	0.4964	0.078*	
H16B	0.3161	0.6923	0.4951	0.078*	
C17	0.4344 (3)	0.72010 (16)	0.77297 (19)	0.0544 (7)	
H17A	0.3647	0.7055	0.8073	0.065*	
C18	0.5624 (3)	0.69034 (16)	0.82958 (19)	0.0534 (7)	
C19	0.6790 (3)	0.6934 (2)	0.7902 (2)	0.0677 (9)	
H19A	0.6775	0.7173	0.7314	0.081*	
C20	0.7921 (3)	0.6614 (2)	0.8381 (2)	0.0739 (9)	
H20A	0.8672	0.6628	0.8117	0.089*	
C21	0.7952 (3)	0.62780 (19)	0.9240 (2)	0.0659 (8)	
C22	0.6914 (3)	0.62547 (17)	0.9676 (2)	0.0685 (9)	
H22A	0.6975	0.6036	1.0279	0.082*	
C23	0.5704 (3)	0.65809 (17)	0.9176 (2)	0.0625 (8)	
H23A	0.4968	0.6571	0.9458	0.075*	
C24	0.4343 (3)	0.80943 (16)	0.76286 (19)	0.0541 (7)	
C25	0.3778 (3)	0.85731 (18)	0.8273 (2)	0.0622 (8)	
H25A	0.3412	0.8334	0.8747	0.075*	
C26	0.3776 (3)	0.93915 (19)	0.8193 (2)	0.0686 (9)	
H26A	0.3419	0.9705	0.8615	0.082*	
C27	0.4299 (3)	0.97249 (18)	0.7496 (2)	0.0639 (8)	
C28	0.4854 (4)	0.93165 (17)	0.6871 (2)	0.0738 (9)	
H28A	0.5213	0.9574	0.6405	0.089*	
C29	0.4875 (3)	0.84671 (16)	0.6948 (2)	0.0694 (9)	
H29A	0.5257	0.8168	0.6527	0.083*	

### Atomic displacement parameters (Å^2^)

**Table:**

	*U*^11^	*U*^22^	*U*^33^	*U*^12^	*U*^13^	*U*^23^
O	0.0676 (13)	0.0462 (12)	0.0759 (14)	0.0018 (10)	−0.0091 (11)	−0.0051 (10)
N1	0.0629 (15)	0.0381 (12)	0.0640 (15)	0.0030 (11)	−0.0082 (12)	−0.0007 (10)
F1	0.0811 (14)	0.1069 (17)	0.0887 (14)	0.0046 (12)	−0.0196 (11)	0.0062 (12)
C1	0.0533 (16)	0.0475 (17)	0.0649 (18)	0.0035 (13)	0.0188 (14)	−0.0039 (13)
F2	0.1064 (16)	0.0490 (11)	0.1124 (16)	0.0025 (10)	−0.0030 (12)	−0.0074 (10)
N2	0.0521 (14)	0.0410 (13)	0.0625 (14)	0.0018 (10)	0.0027 (11)	−0.0030 (11)
C2	0.069 (2)	0.055 (2)	0.090 (2)	−0.0001 (16)	0.0066 (18)	−0.0102 (17)
C3	0.085 (3)	0.065 (2)	0.137 (4)	−0.009 (2)	−0.004 (3)	−0.022 (2)
C4	0.108 (3)	0.047 (2)	0.159 (4)	−0.004 (2)	−0.004 (3)	−0.011 (2)
C5	0.087 (3)	0.051 (2)	0.124 (3)	0.0107 (19)	0.007 (2)	−0.001 (2)
C6	0.0568 (18)	0.0496 (17)	0.079 (2)	0.0041 (14)	0.0173 (16)	−0.0012 (15)
C7	0.0619 (19)	0.0530 (19)	0.081 (2)	0.0157 (15)	0.0110 (17)	0.0063 (15)
C8	0.0539 (18)	0.062 (2)	0.0681 (19)	0.0056 (14)	0.0009 (15)	−0.0024 (15)
C9	0.0589 (17)	0.0467 (17)	0.0542 (16)	0.0052 (13)	0.0094 (14)	−0.0032 (13)
C10	0.0531 (16)	0.0460 (16)	0.0564 (16)	0.0065 (13)	0.0065 (13)	−0.0008 (13)
C11	0.078 (2)	0.0483 (18)	0.0691 (19)	0.0024 (16)	−0.0140 (16)	−0.0005 (14)
C12	0.086 (2)	0.0466 (17)	0.067 (2)	−0.0017 (16)	−0.0093 (17)	0.0013 (14)
C13	0.0586 (17)	0.0437 (16)	0.0726 (19)	0.0031 (13)	0.0000 (15)	−0.0022 (14)
C14	0.0564 (17)	0.0428 (16)	0.0652 (18)	0.0035 (13)	−0.0052 (14)	0.0007 (13)
C15	0.0629 (19)	0.0409 (15)	0.080 (2)	0.0054 (13)	0.0041 (16)	−0.0024 (14)
C16	0.071 (2)	0.0421 (16)	0.073 (2)	0.0080 (14)	−0.0099 (16)	0.0003 (14)
C17	0.0578 (17)	0.0530 (17)	0.0549 (17)	−0.0052 (13)	0.0171 (14)	−0.0029 (13)
C18	0.0648 (18)	0.0462 (16)	0.0502 (16)	−0.0079 (13)	0.0130 (14)	−0.0071 (12)
C19	0.064 (2)	0.088 (2)	0.0504 (17)	−0.0104 (17)	0.0069 (15)	0.0033 (16)
C20	0.0564 (19)	0.102 (3)	0.060 (2)	−0.0097 (18)	0.0020 (15)	−0.0002 (18)
C21	0.067 (2)	0.064 (2)	0.0599 (19)	−0.0063 (16)	−0.0058 (17)	−0.0075 (16)
C22	0.098 (3)	0.0520 (18)	0.0523 (17)	−0.0059 (17)	0.0052 (18)	0.0012 (14)
C23	0.081 (2)	0.0505 (18)	0.0587 (18)	−0.0065 (16)	0.0211 (16)	−0.0028 (14)
C24	0.0537 (16)	0.0503 (17)	0.0581 (17)	−0.0044 (13)	0.0094 (13)	−0.0080 (13)
C25	0.0598 (18)	0.067 (2)	0.0615 (18)	0.0012 (15)	0.0144 (14)	−0.0040 (15)
C26	0.066 (2)	0.062 (2)	0.075 (2)	0.0126 (16)	0.0056 (17)	−0.0190 (17)
C27	0.067 (2)	0.0490 (18)	0.069 (2)	0.0003 (15)	−0.0056 (16)	−0.0108 (15)
C28	0.099 (3)	0.0538 (19)	0.068 (2)	−0.0155 (18)	0.0136 (19)	−0.0032 (16)
C29	0.095 (2)	0.0533 (18)	0.0664 (19)	−0.0074 (17)	0.0318 (18)	−0.0123 (15)

### Geometric parameters (Å, º)

**Table:**

O—C9	1.373 (3)	C13—C14	1.508 (4)
O—C11	1.434 (3)	C13—H13A	0.9700
N1—C12	1.461 (4)	C13—H13B	0.9700
N1—C13	1.465 (3)	C14—H14A	0.9700
N1—C16	1.468 (3)	C14—H14B	0.9700
F1—C21	1.370 (4)	C15—C16	1.492 (4)
C1—C6	1.408 (4)	C15—H15A	0.9700
C1—C2	1.408 (4)	C15—H15B	0.9700
C1—C10	1.417 (4)	C16—H16A	0.9700
F2—C27	1.371 (3)	C16—H16B	0.9700
N2—C14	1.461 (3)	C17—C18	1.513 (4)
N2—C15	1.465 (3)	C17—C24	1.514 (4)
N2—C17	1.474 (3)	C17—H17A	0.9800
C2—C3	1.364 (5)	C18—C23	1.360 (4)
C2—H2A	0.9300	C18—C19	1.431 (3)
C3—C4	1.373 (5)	C19—C20	1.361 (4)
C3—H3A	0.9300	C19—H19A	0.9300
C4—C5	1.353 (5)	C20—C21	1.349 (4)
C4—H4A	0.9300	C20—H20A	0.9300
C5—C6	1.410 (4)	C21—C22	1.344 (4)
C5—H5A	0.9300	C22—C23	1.439 (3)
C6—C7	1.411 (4)	C22—H22A	0.9300
C7—C8	1.354 (4)	C23—H23A	0.9300
C7—H7A	0.9300	C24—C29	1.360 (4)
C8—C9	1.408 (4)	C24—C25	1.431 (3)
C8—H8A	0.9300	C25—C26	1.385 (4)
C9—C10	1.360 (4)	C25—H25A	0.9300
C10—H10A	0.9300	C26—C27	1.345 (4)
C11—C12	1.511 (4)	C26—H26A	0.9300
C11—H11A	0.9700	C27—C28	1.341 (4)
C11—H11B	0.9700	C28—C29	1.437 (3)
C12—H12A	0.9700	C28—H28A	0.9300
C12—H12B	0.9700	C29—H29A	0.9300
			
C9—O—C11	116.7 (2)	N2—C14—H14B	109.5
C12—N1—C13	111.5 (2)	C13—C14—H14B	109.5
C12—N1—C16	110.0 (2)	H14A—C14—H14B	108.1
C13—N1—C16	108.5 (2)	N2—C15—C16	110.7 (2)
C6—C1—C2	118.6 (3)	N2—C15—H15A	109.5
C6—C1—C10	119.4 (3)	C16—C15—H15A	109.5
C2—C1—C10	122.0 (3)	N2—C15—H15B	109.5
C14—N2—C15	106.8 (2)	C16—C15—H15B	109.5
C14—N2—C17	113.1 (2)	H15A—C15—H15B	108.1
C15—N2—C17	111.6 (2)	N1—C16—C15	111.2 (2)
C3—C2—C1	120.5 (3)	N1—C16—H16A	109.4
C3—C2—H2A	119.8	C15—C16—H16A	109.4
C1—C2—H2A	119.8	N1—C16—H16B	109.4
C2—C3—C4	120.8 (4)	C15—C16—H16B	109.4
C2—C3—H3A	119.6	H16A—C16—H16B	108.0
C4—C3—H3A	119.6	N2—C17—C18	110.8 (2)
C5—C4—C3	120.6 (4)	N2—C17—C24	110.2 (2)
C5—C4—H4A	119.7	C18—C17—C24	111.5 (2)
C3—C4—H4A	119.7	N2—C17—H17A	108.1
C4—C5—C6	120.8 (3)	C18—C17—H17A	108.1
C4—C5—H5A	119.6	C24—C17—H17A	108.1
C6—C5—H5A	119.6	C23—C18—C19	117.8 (3)
C1—C6—C5	118.7 (3)	C23—C18—C17	121.8 (2)
C1—C6—C7	118.5 (3)	C19—C18—C17	120.3 (2)
C5—C6—C7	122.8 (3)	C20—C19—C18	120.4 (3)
C8—C7—C6	121.4 (3)	C20—C19—H19A	119.8
C8—C7—H7A	119.3	C18—C19—H19A	119.8
C6—C7—H7A	119.3	C21—C20—C19	120.0 (3)
C7—C8—C9	119.9 (3)	C21—C20—H20A	120.0
C7—C8—H8A	120.1	C19—C20—H20A	120.0
C9—C8—H8A	120.1	C22—C21—C20	123.3 (3)
C10—C9—O	125.3 (2)	C22—C21—F1	117.8 (3)
C10—C9—C8	120.6 (3)	C20—C21—F1	119.0 (3)
O—C9—C8	114.1 (2)	C21—C22—C23	117.6 (3)
C9—C10—C1	120.2 (3)	C21—C22—H22A	121.2
C9—C10—H10A	119.9	C23—C22—H22A	121.2
C1—C10—H10A	119.9	C18—C23—C22	120.8 (3)
O—C11—C12	109.6 (2)	C18—C23—H23A	119.6
O—C11—H11A	109.8	C22—C23—H23A	119.6
C12—C11—H11A	109.8	C29—C24—C25	118.0 (3)
O—C11—H11B	109.8	C29—C24—C17	122.4 (2)
C12—C11—H11B	109.8	C25—C24—C17	119.6 (3)
H11A—C11—H11B	108.2	C26—C25—C24	120.3 (3)
N1—C12—C11	114.4 (3)	C26—C25—H25A	119.9
N1—C12—H12A	108.7	C24—C25—H25A	119.9
C11—C12—H12A	108.7	C27—C26—C25	118.9 (3)
N1—C12—H12B	108.7	C27—C26—H26A	120.6
C11—C12—H12B	108.7	C25—C26—H26A	120.6
H12A—C12—H12B	107.6	C28—C27—C26	124.3 (3)
N1—C13—C14	111.7 (2)	C28—C27—F2	117.1 (3)
N1—C13—H13A	109.3	C26—C27—F2	118.6 (3)
C14—C13—H13A	109.3	C27—C28—C29	117.6 (3)
N1—C13—H13B	109.3	C27—C28—H28A	121.2
C14—C13—H13B	109.3	C29—C28—H28A	121.2
H13A—C13—H13B	107.9	C24—C29—C28	120.9 (3)
N2—C14—C13	110.5 (2)	C24—C29—H29A	119.5
N2—C14—H14A	109.5	C28—C29—H29A	119.5
C13—C14—H14A	109.5		
			
C6—C1—C2—C3	0.7 (5)	C13—N1—C16—C15	−55.9 (3)
C10—C1—C2—C3	179.8 (3)	N2—C15—C16—N1	60.9 (3)
C1—C2—C3—C4	0.1 (6)	C14—N2—C17—C18	51.5 (3)
C2—C3—C4—C5	−0.2 (7)	C15—N2—C17—C18	171.9 (2)
C3—C4—C5—C6	−0.5 (7)	C14—N2—C17—C24	175.4 (2)
C2—C1—C6—C5	−1.4 (4)	C15—N2—C17—C24	−64.1 (3)
C10—C1—C6—C5	179.5 (3)	N2—C17—C18—C23	−125.1 (3)
C2—C1—C6—C7	178.7 (3)	C24—C17—C18—C23	111.7 (3)
C10—C1—C6—C7	−0.4 (4)	N2—C17—C18—C19	53.0 (3)
C4—C5—C6—C1	1.3 (6)	C24—C17—C18—C19	−70.1 (3)
C4—C5—C6—C7	−178.8 (4)	C23—C18—C19—C20	3.0 (4)
C1—C6—C7—C8	−1.7 (5)	C17—C18—C19—C20	−175.2 (3)
C5—C6—C7—C8	178.4 (3)	C18—C19—C20—C21	−1.1 (5)
C6—C7—C8—C9	1.9 (5)	C19—C20—C21—C22	−1.7 (5)
C11—O—C9—C10	0.5 (4)	C19—C20—C21—F1	177.2 (3)
C11—O—C9—C8	−179.8 (3)	C20—C21—C22—C23	2.4 (5)
C7—C8—C9—C10	0.1 (5)	F1—C21—C22—C23	−176.5 (2)
C7—C8—C9—O	−179.7 (3)	C19—C18—C23—C22	−2.3 (4)
O—C9—C10—C1	177.5 (2)	C17—C18—C23—C22	175.9 (3)
C8—C9—C10—C1	−2.2 (4)	C21—C22—C23—C18	−0.3 (4)
C6—C1—C10—C9	2.3 (4)	N2—C17—C24—C29	−40.4 (4)
C2—C1—C10—C9	−176.8 (3)	C18—C17—C24—C29	83.1 (3)
C9—O—C11—C12	−172.2 (3)	N2—C17—C24—C25	140.3 (3)
C13—N1—C12—C11	89.7 (3)	C18—C17—C24—C25	−96.2 (3)
C16—N1—C12—C11	−149.9 (3)	C29—C24—C25—C26	0.3 (4)
O—C11—C12—N1	−64.6 (4)	C17—C24—C25—C26	179.6 (3)
C12—N1—C13—C14	176.5 (2)	C24—C25—C26—C27	0.7 (4)
C16—N1—C13—C14	55.2 (3)	C25—C26—C27—C28	−1.3 (5)
C15—N2—C14—C13	60.2 (3)	C25—C26—C27—F2	179.1 (3)
C17—N2—C14—C13	−176.6 (2)	C26—C27—C28—C29	0.8 (5)
N1—C13—C14—N2	−59.4 (3)	F2—C27—C28—C29	−179.6 (3)
C14—N2—C15—C16	−61.4 (3)	C25—C24—C29—C28	−0.8 (5)
C17—N2—C15—C16	174.6 (2)	C17—C24—C29—C28	179.9 (3)
C12—N1—C16—C15	−178.1 (3)	C27—C28—C29—C24	0.3 (5)
